# Prognosis of triple-negative breast cancer associated with pregnancy: A propensity score-matched analysis from the French CALG (Cancer Associé à la Grossesse) network

**DOI:** 10.1016/j.breast.2022.01.004

**Published:** 2022-01-10

**Authors:** Anne Puchar, Marie Despierres, Anne-Sophie Boudy, Lise Selleret, Joseph Gligorov, Sandrine Richard, Sonia Zilberman, Clément Ferrier, Yohann Dabi, Valentin Varlas, Isabelle Thomassin-Naggara, Sofiane Bendifallah, Cyril Touboul, Emile Darai

**Affiliations:** a Department of Gynaecology and Obstetrics, Tenon University Hospital, Assistance Publique des Hôpitaux de Paris (AP-HP), Sorbonne University, France; b Cancer Associé à La Grossesse (CALG), French CALG Network, France; c UMRS-938 4. Faculté́ de Médecine Sorbonne Université́, France; d Department of Oncology, Tenon University Hospital, Assistance Publique des Ho^pitaux de Paris (AP-HP), Sorbonne University, France; e Department of Radiology, Tenon University Hospital, Assistance Publique des Ho^pitaux de Paris (AP-HP), Sorbonne University, France

**Keywords:** Pregnancy-associated breast cancer, Pregnancy, Triple-negative breast cancer, Prognosis, Breast cancer-free survival, TNBC, triple-negative breast cancer, TN-PABC, triple-negative pregnancy associated breast cancer, PPBC, Post-partum breast cancer, CALG, Cancer Associé à la Grossesse, INCa, Institut National du Cancer, PS, Propensity score, PSM, propensity matching procedures, OS, overall survival, RFS, recurrence-free survival

## Abstract

**Introduction:**

Triple-negative (TN) breast cancer represents one third of pregnancy-associated breast cancers (PABC). The aims of the current study were to describe oncological and obstetrical outcomes of patients with TN-PABC and to compare their prognosis with TN-non-PABC patients using a propensity score.

**Materials and methods:**

Between January 2005 and December 2020, data of patients with histologically proven TN-PABC were collected and compared with data of TN-non-PABC patients under the age of 46 years diagnosed during the same period using a propensity score (PS).

**Results:**

After PS matching (tumor size and lymph node involvement),there were 59 patients in each group. The median follow-up was 14 months (IQR 4.8–40.1) for the TN-PABC group and 60 months (IQR 30.7–101.4) for the TN-non-PABC group. Eight recurrences occurred in the TN-PABC group and 10 in the TN-non-PABC group (adjusted OR (AOR) = 0.60 (0.21–1.60), HR (Cox adjusted model- AHR) = 1.25 (0.53–2.94)). Two patients died in the TN-PABC group, and six in the TN-non-PABC group with an AOR = 0.23 (0.03–1.01) and an AHR = 0.58 (0.12–2.69). All the patients diagnosed during the second (n = 17) and third trimesters (n = 28) continued their pregnancies, with a median term at delivery of 38 WG (IQR 36–39). All patients gave birth to healthy newborns.

**Conclusion:**

Although the TN subtype is associated with poor prognosis in pregnant patients due to advanced stage at diagnosis and high lymph node involvement, our PS-matched case-control study showed that pregnancy per se does not worsen the prognosis in terms of recurrence-free and overall survival.

## Highlights

• Triple-negative breast cancer are associated with poor prognosis.
• Breast cancers occurring during pregnancy differ from those occurring in the post-partum period.
• Pregnancy per se does not worsen the prognosis in triple-negative breast cancer.

## 1 Introduction

An estimated that 2.3 million women were diagnosed with breast cancer in 2020 representing about one-quarter of all cancers worldwide and causing 685,000 deaths [1]. A late age at first full-term pregnancy is an important risk factor of breast cancer especially in developed countries [2,3]. This explains why breast cancer is also the most common pregnancy-associated cancer with an incidence of 1/3000 [4,5] representing approximately 4–7% of breast cancers diagnosed in women under 45 years [6,7]. This rate is on the increase as more women delay childbearing [4,6,[8], [9], [10], [11], [12]].

Breast cancer can be divided into four different subtypes, as described by Perou et al. depending on a 50-gene expression signature (PAM50): luminal A and luminal B (expressing the estrogen receptor (ER)), basal-like and human epidermal growth factor receptor 2 (HER2)-enriched without ER expression [13]. In clinical practice, a surrogate classification based on histology and immunochemistry is used defining 5 sub-types: triple negative, HER2-enriched non luminal, luminal B-like HER2+, Luminal B-like HER2-and luminal A like [14].

In a large analysis of the Surveillance, Epidemiology and End-Results (SEER) database including 196,094 patients, Howlader et al. showed that the triple-negative breast cancer (TNBC) subtype represented 9.7% of all cases and was associated with the lowest cancer-specific survival regardless of stage at diagnosis [15].

Pregnancy-associated cancers are defined as cancers diagnosed during pregnancy or within the year following delivery. However, several studies distinguished breast cancers occurring during pregnancy (Pregnancy associated breast cancer: PABC) from those occurring in the post-partum period (Post-partum breast cancer: PPBC). Indeed, the latest are associated with worse survival rates and increased risk of metastasis than breast cancers diagnosed in young pregnant or nulliparous women [16,17], which could be explained by immunosuppressive and wound healing-like alterations in post-partum involuting breast [18]. Therefore, in a recent publication, Amant et al. recommend both entities to be considered separately [19].

There is evidence suggesting that tumors diagnosed during pregnancy and around delivery exhibit adverse prognostic characteristics. Indeed, in a cohort of women diagnosed with invasive breast cancer between 1992 and 2009 in Sweden, Johansson et al. reported higher proportions of ER/PR negative, HER 2 positive and triple-negative tumors in women with PABC compared to nulliparous women [20]. Their results were consistent with those published by Amant et al. in a multicentre cohort-study including 311 pregnant patients and 865 nonpregnant patients [17].

Although a meta-analysis of 30 studies concluded that women with PABC had a poorer prognosis than non-PABC patients even after adjustment for confounding factors, national and international guidelines recommend that PABC treatment should be as similar as possible to that in non-PABC patients [21]. However, few data are available about the management and survival of women with TNBC associated with pregnancy (TN-PABC). Therefore, the aims of the current study were to describe oncological and obstetrical outcomes of patients with TN-PABC and to compare their prognosis and survival with TNBC not associated with pregnancy (TN-non-PABC) patients using a propensity score (PS) analysis.

## 2 Material and methods

This was a retrospective analysis from the prospective database of the French Pregnancy-Associated Cancer Network (Cancer Associé à la Grossesse - CALG) created by the Institut National du Cancer (INCa) (Tenon University Hospital, Paris, France) and collates cases of cancers associated with pregnancy (diagnosed during pregnancy or during the first post-partum year).

### 2.1 Study population

Between January 2005 and December 2020, data of patients with histologically proven TN-PABC were identified (TN-PABC group) from the CALG database. Patients diagnosed during the first postpartum year were excluded from analysis. Data of all non-pregnant patients with histologically proven TNBC under the age of 46 years and diagnosed between January 2005 and December 2020 were identified from the Tenon Hospital database to form the TN-non-PABC group for comparison. The age of 46 years was set as the threshold as it corresponded to the oldest patient with TN-PABC in our cohort. Women with multifocal cancers were excluded from both groups.

The Ethics Committee (CEROG) of the Collège National des Gynécologues et Obstétriciens Français (CNGOF) approved the study (CEROG 2019-GYN-603).

### 2.2 Data collection

The following data were recorded: epidemiological data (age and parity at diagnosis, genetic mutation, familial or personal history of cancer, term at diagnosis); and type of cancer and histological details (histological grade according to Ellis and Easton, HR status (ER and PR), HER2 overexpression, tumor size, Ki67 expression). Diagnosis was systematically proven by histology. The histological data corresponded to surgical specimens, except when neoadjuvant chemotherapy was performed in which case biopsy data and initial imaging data were used. For patients undergoing neoadjuvant chemotherapy, lymph node status was determined by axillary lymph node cytology before chemotherapy and on analysis of a surgical specimen using the Residual Cancer Burden (RCB) index [22]. A Ki67 threshold of >15% was used to denote a proliferative tumor, though analysis was also performed with a Ki67 ≥ 20% as this cut-off is routinely used in France. ER and/or PR status was considered to be negative when <10%, but we also screened patients with a HR status <1%. If HER2 expression was moderate (score 2), a fluorescent in situ hybridization test (FISH) was performed.

Disease stage (TNM classification), treatments during pregnancy and after delivery, follow-up, term and mode of delivery, and neonatal and maternal outcomes were recorded.

Treatment modalities for TN-PABC followed the guidelines and were discussed in multidisciplinary meetings. Patient status was determined on December 31, 2020 by reviewing the patients’ charts or by requesting follow-up data from the treating physicians if the patients were treated elsewhere.

Recurrent disease was assessed by physical examination, histological findings, clinical follow-up, and imaging. Recurrence-free survival (RFS) was defined as time from diagnosis to breast cancer recurrence and was censored at the date of the last follow-up or at the date of death without recurrence. In our cohort, overall survival (OS) was defined as time from diagnosis to breast cancer-related death. Recurrences were defined as: i) local if recurrence was ipsilateral, ii) regional if ipsilateral axillary recurrence, iii) distant if metastasis to bone, liver, lung, brain, or peritoneum, and for contralateral axillary recurrence.

### 2.3 Statistical analysis

#### 2.3.1 Bivariate analysis

The two groups, TN-PABC and TN-non-PABC, were compared in terms of demographics and treatment characteristics, before PS matching (PSM).

Categorical variables are presented as the number of cases and percentages, while continuous variables are presented as the median and interquartile range (IQR).

TN characteristics were compared between the PABC and non-PABC patients using bivariate analysis to select potential confounding factors (i.e., those with a p-value <20%). Continuous variables were compared using the Student's t-test. Chi2 or Fisher tests were applied to assess the relationship between categorical variables.

#### 2.3.2 Propensity score (PS) and matching procedures (PSM)

As this was a small population and in order to be free of any bias that could negatively impact survival and recurrent disease outcome in the two groups, we performed a multivariate analysis to make the two populations comparable. A PS was then generated using a logistic regression model as described by Rosenbaum and Rubin [23,24]. The variables included in the PS were the variables associated with death and recurrence.

Each woman of the TN-PABC group was matched (1:1 match) to a corresponding woman in the TN-non-PABC group using the algorithm of the nearest neighbor matching. OS and RFS were estimated, first, by conditional logistic regression with matching on the PS reported as adjusted odds ratios (ORs) and 95% confidence intervals (CIs); then, by a Cox proportional hazards model matching on the reported hazard ratios (HRs) and 95% CIs.

All statistical analyses were performed with RStudio version 4.0.3 [2020-10-10] software.

## 3 Results

### 3.1 Characteristics of the population

During the study period, 301 patients with PABC were extracted from the CALG network database of Tenon University Hospital. Among these, 69 patients were excluded because breast cancer was diagnosed during the postpartum period, and 169 because of non-TN-PABC. The rate of TN-PABC was 27.1% (63/232 patients). Among the 63 remaining patients, we excluded four patients presenting multifocal cancer to avoid treatment biases. Therefore, 59 TN-PABC patients were retained for analysis. Ninety-two women were identified in the Tenon Hospital database to form the TN-non-PABC group, as previously described.

The epidemiological and histological characteristics of the population before PSM are summarized in Table 1.

**Table 1:** Characteristics of the population and results of the bivariate analysis between pregnancy and the other variables.

	Before PSM			After PSM				
	TN-Non-PABC group N = 92	TN-PABC N = 59	Bivariate analysis		TN-Non-PABC group N = 59	TN-PABC group N = 59	p-value	
Age - years median (IQR)	38.5 (33.8–42.0)	33.8 (30.0–37.0)	1.4.10^−5^		35.7 (32.0–39.0)	33.8 (30.0–37.0)	**0.03**	
Term at diagnosis (WG)	–	28.0 (15.6–31.0)	0.21		–	28 (17.5–31.0)	0.07	
BMI - kg/m^2^ median (IQR)	24.3 (22.0–27.3)	27.3 (23.9–30.8)	0.17		22.0 (21.0–25.0)	27.3 (23.9–30.8)	0.74	
Parity - median (IQR)	1.0 (0.0–2.0)	1.0 (0.0–2.0)	0.19		1.0 (0.0–2.0)	1.0 (0.0–2.0)		
Mutation - N (%)								
No	43 (46.7)	13 (22.0)	0.077		28 (47.5)	13 (22.0)	0.27	
Yes	17 (18.5)	10 (17.0)	0.0079		10 (16.9)	10 (16.9)		
NA	32 (34.8)	36 (61.0)	0.0013		21 (35.6)	36 (61.0)		
Personal history of cancer – N (%)								
No	91 (98.9)	55 (93.2)	0.53		59 (100)	55 (93.2)	**0.04**	
Yes	1 (1.1)	4 (6.8)	0.39		0	4 (6.8)		
Cancer stage- N (%)								
T								
1	22 (23.9)	7 (11.9)	0.52		12 (20.3)	7 (11.9)	0.06	
2	48 (52.2)	26 (44.1)	0.60		34 (57.6)	26 (44.1)		
3	9 (9.8)	18 (30.5)	0.16		7 (11.9)	18 (30.5)		
4	13 (14.1)	8 (13.5)	0.43		6 (10.2)	8 (13.5)		
N								
0	56 (60.9)	28 (47.5)	0.51		35 (59.3)	28 (47.5)	0.20	
1	25 (27.2)	31 (52.5)	0.13		24 (40.7)	31 (52.5)		
2	5 (5.4)	0			0	0		
3	6 (6.5)	0			0	0		
M								
0	84 (91.3)	56 (94.9)			56 (94.9)	56 (94.9)	1	
1	8 (8.7)	3 (5.1)			3 (5.1)	3 (5.1)		
Histological type - N (%)								
Ductal	92 (100)	58 (98.3)			59 (100)	58 (98.3)	1	
Lobular	0	1 (1.7)			0	1 (1.7)		
Grade –N (%)								
Low	0	0			0	0		
Moderate	11 (12.0)	9 (15.3)			9 (15.3)	9 (15.3)	0.36	
High	80 (87.0)	48 (81.4)			50 (84.7)	48 (81.4)		
NA	1 (1.0)	2 (3.4)			0	2 (3.4)		
HR ≤ 1% N (%)								
No	23 (25.0)	17 (28.8)			10 (16.9)	17 (28.8)	0.13	
Yes	69 (75.0)	42 (71.2)			49 (83.1)	42 (71.2)		
Ki67 ≥ 15% - N (%)								
No	1 (1.1)	3 (5.3)			1 (1.7)	3 (5.3)	0.29	
Yes	91 (98.9)	54 (94.7)			58 (98.3)	54 (94.7)		
Ki67 > 20% N (%)								
No	3 (3.3)	4 (7.0)			3 (5.1)	4 (7.0)	0.66	
Yes	89 (96.7)	53 (93.0)			56 (94.9)	53 (93.0)		
Breast cancer recurrence – N (%)								
No	75 (81.5)	51 (86.4)			49 (83.1)	51 (86.4)	0.61	
Yes	17 (18.5)	8 (13.6)			10 (16.9)	8 (13.6)		
Death – N (%)								
No	82 (89.1)	57 (96.6)			53 (89.8)	57 (96.6)	0.14	
Yes	10 (10.9)	2 (3.4)			6 (10.2)	2 (3.4)		
Follow-up (days) – Median (IQR)	1478 (556.5–2483.0)	396 (144.5–1204.0)			1789 (919.5–3042.5)	396 (144.5–1204)

**Table 2:** Treatments received in patients with TN-PABC and TN-non-PABC group after PSM.

	TN-PABC group (n = 59)	TN-non-PABC group (n = 59)	p
Chemotherapy n (%)			
Neoadjuvant	39 (66.1)	30 (50.7)	
Adjuvant	15 (25.4)	20 (33.8)	
Neoadjuvant & Adjuvant	5 (8.5)	9 (15.5)	0.23
Mammary surgery n (%)			
Partial mastectomy	31 (52.5)	36 (61.0)	
Total mastectomy	25 (42.4)	22 (37.3)	
None	3 (5.1)	3 (5.1)	0.77
Axillary surgery, n (%)			
Sentinel lymph node (SLN)	16 (27.1)	29 (49.2)	
Lymphadenectomy	40 (67.8)	30 (50.8)	
None	5 (8.4)	3 (5.1)	0.06
Postpartum radiotherapy n (%)	51 (86.4)	52 (88.1)	1

Median age at diagnosis was significantly higher in the TN-non-PABC group (38.5 years – IQR 33.8–42.0) than in the TN-PABC group (33.8 years – IQR 30.0–37.0) (p < 0.05). Lower tumor size (T) (p < 0.05) and less lymph node involvement (N) (p < 0.05) were observed in the TN-non-PABC group compared to the TN-PABC group.

In the TN-PABC group, the median tumor size was 45.5 mm (IQR 31.5–60.75), 26 (44.1%) patients had a tumor classified T2, 18 (30.5%) a tumor classified T3, and eight (13.5%) a tumor classified T4. In the TN-non-PABC group, the median tumor size was 30 mm (IQR 21.0–46.5), and almost three quarters of the patients had a tumor classified T2 or less.

Fifty-two percent of the TN-PABC group had nodal involvement (n = 31), while nodal involvement was found in 39.1% (n = 36) in TN-non-PABC group, p < 0.05.

No significant differences in BMI (p = 0.21), parity (p = 0.17), genetic mutation (p = 0.19), distant metastasis (p = 0.53), tumor type (p = 0.39), tumor grade (p = 0.52) or Ki67 (p = 0.16 and 0.43) were found between the groups. In both groups, most of the tumors were aggressive and of high grade: 81.4% and 87% of the women had high grade in the TN-PABC and TN-non-PABC groups, respectively (p = 0.67); and 94.7% and 98.9%, respectively, had a Ki67 ≥ 15% (p = 0.20).

As tumor size and lymph node involvement were significantly associated with death and recurrence, these two covariates were included in the PS model to adjust and optimize the matching procedure.

One hundred and eighteen women (59 patients in each group) were retained after matching with the PS model. All the TN-PABC women were matched which did not invalidate the model. After PSM, the two groups were comparable for tumor size (p = 0.06) and lymph node status (p = 0.20).

### 3.2 Characteristics of the TN-PABC group

Median term at diagnosis was 28 weeks of gestation (WG) (IQR 15.6–31.0). Thirteen patients (22%) were diagnosed during the 1st trimester, 17 (28.8%) during the 2nd trimester and 28 (47.5%) during the 3rd trimester. Chemotherapy was always delivered after 14 WG.

Among the 13 patients diagnosed during the 1st trimester, two (15.4%) had a spontaneous miscarriage, one (7.7%) an abortion, five (38.5%) a medical termination of pregnancy and three (23.1%) continued their pregnancies. Data on pregnancy outcome was missing for two patients.

All the patients diagnosed during the second (n = 17) and third trimesters (n = 28) continued their pregnancies, with a median term at delivery of 38 WG (IQR 36–39). Seven out of 48 patients (14.6%) gave birth prematurely. All patients gave birth to healthy newborns.

### 3.3 Treatments received (Table 2)

Chemotherapy management did not significantly differ between the groups (p = 0.23). In the TN-PABC group, 66.1% of patients were given neoadjuvant chemotherapy versus 50.7% in the TN-non-PABC group. Fifteen out of 59 patients (25.4%) and 20 out of 59 (33.8%) received adjuvant chemotherapy in TN-PABC group and TN-non-PABC group respectively; 8.5% of patients in TN-PABC group and 15.5% of patients in TN-non-PABC group both had neoadjuvant and adjuvant chemotherapy. Median term at the first course of chemotherapy was 30 WG (IQR 20–32.3). The chemotherapy schedule for the TN-PABC depended on the term at diagnosis. For each patient, no chemotherapy was administered after 35 WG. In both groups, the chemotherapy regimen included anthracyclines (adriamycin or epirubicine), taxanes, and cyclophosphamide. Patients in the TN-non-PABC group received dose-dense chemotherapy.

No differences were found between the groups concerning the type of surgery (partial or total mastectomy) or radiotherapy modalities (p = 0.77 and p = 1, respectively).

A sentinel lymph node procedure was performed in 16/59 patients (27.1%) in the TN-PABC group versus 29/59 (49.2%) in the TN-non-PABC group. Axillary lymphadenectomy was more frequent in the TN-PABC group (40/59 (67.8%) versus 30/59 patients (50.8%) in the TN-non-PABC group). A trend for a higher rate of axillary surgery was noted in the TN-PABC group (p = 0.06).

### 3.4 Survival

The median follow-up was 14 months (IQR 4.8–40.1) for the TN-PABC group and 60 months (IQR 30.7–101.4) for the TN-non-PABC group.

### 3.5 Recurrence-free survival

Eight recurrences (13.6%) occurred in the TN-PABC group and 10 (16.9%) in the TN-non-PABC group (adjusted OR = 0.60 (0.21–1.60))

**Fig. 1:**
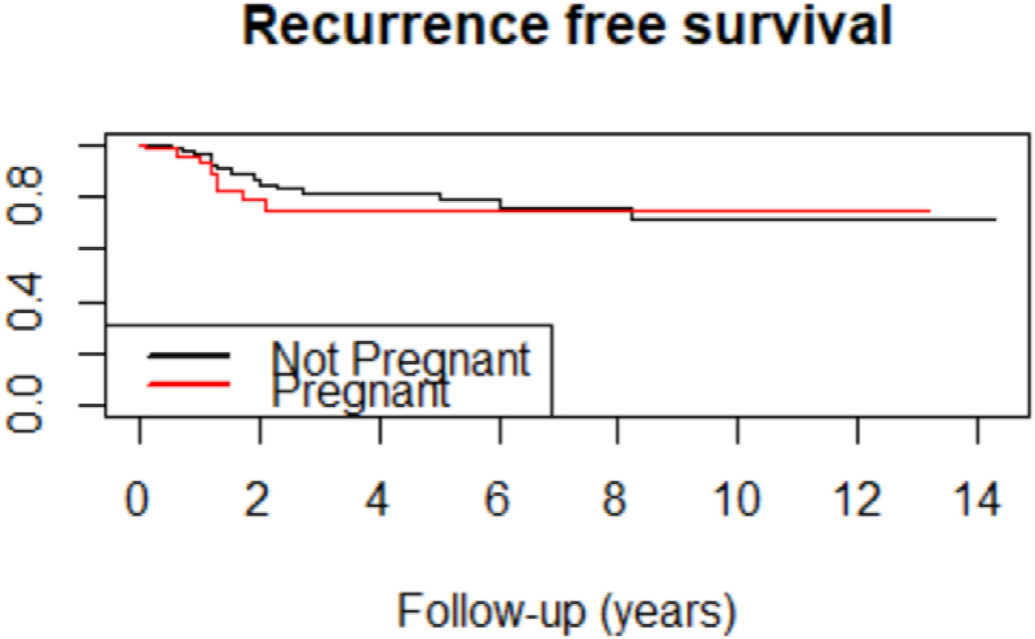
Recurrence-free survival of the TN-PABC and TN-non-PABC groups.

Using the Cox proportional hazards model to take into account the time dimension, RFS was similar in the two groups (Fig. 1 – HR (Cox adjusted model) = 1.25 (0.53–2.94)).

#### 3.5.1 Overall survival

Two (3.4%) patients in the TN-PABC group died, and six (10.2%) in the TN-non-PABC group with an adjusted OR = 0.23 (0.03–1.01).

**Fig. 2:**
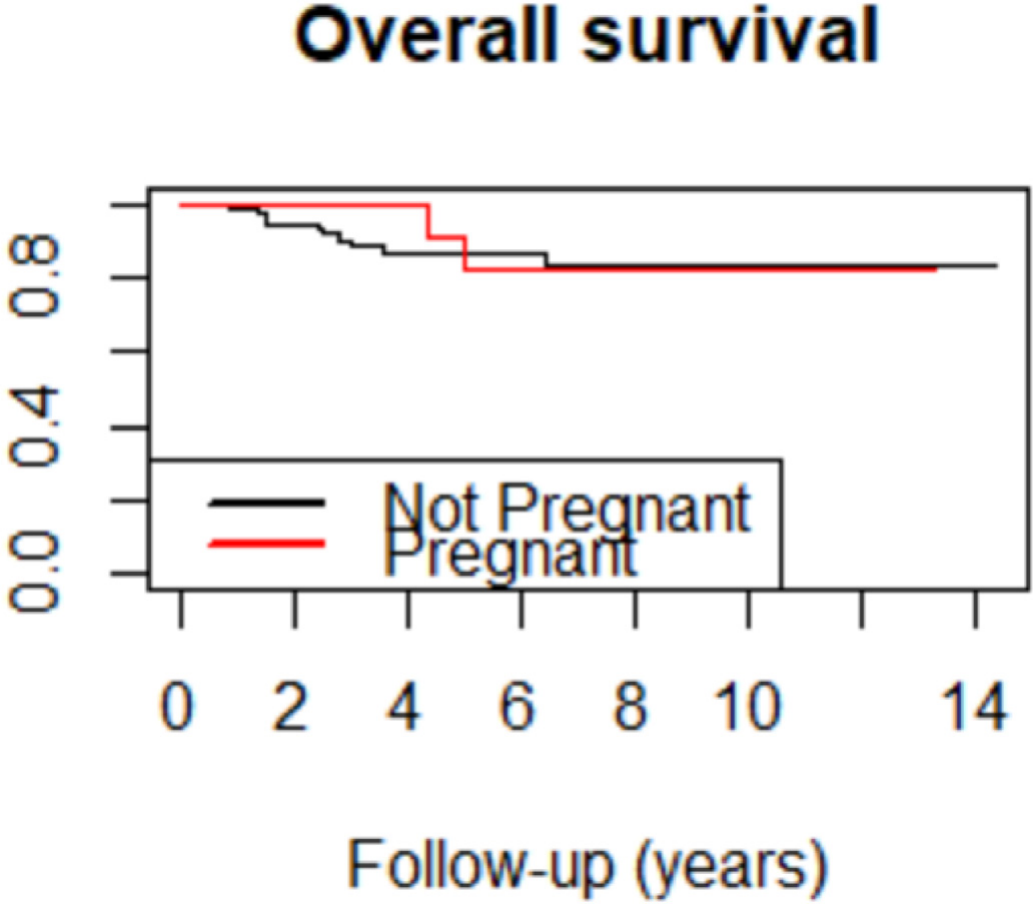
Overall survival of the TN-PABC and TN-non-PABC groups.

Using the Cox proportional hazards model, OS was similar in the two groups (Fig. 2 – HR (Cox adjusted model) = 0.58 (0.12–2.69).

## 4 Discussion

In the current study, TN tumors represented more than one-quarter of the PABCs highlighting the need for specific guidance for mother and fetus management in this setting. Nevertheless, although the TN-PABCs were diagnosed at more advanced stages than the TN-non-PABCs, no differences were found in RFS and OS between the two groups after PSM analysis, suggesting that both populations should be managed in the same way.

Our survival rates are in agreement with those published by Amant et al. who showed that survivals were similar in an international cohort study comparing 311 women with PABC to 865 women with non-PABC without using PSM analysis. After adjusting for age, stage, grade, HR status, HER2 status, histology, type of chemotherapy, use of trastuzumab, radiotherapy, and hormone therapy, both DFS and OS were similar in both groups of patients [17]. Nevertheless, it is important to note that the study period was long and that diagnostic tools and treatment modalities differed widely from country to country. A recent meta-analysis published in 2020 by Shao et al. collecting data from 76 studies showed that PABC was associated with poor prognosis for OS, DFS and cause-specific survival (CSS) [21]. The pooled HRs with 95% CIs for OS, DFS and CSS were 1.45 (1.30–1.63), 1.39 (1.25–1.54) and 1.40 (1.17–1.68), respectively [21]. However, gestational age was not taken into account in this meta-analysis, and no specific analysis was made between patients with true PABC and patients diagnosed in the postpartum period [21]. Moreover, Shao et al. found a non-linear association between the time from last delivery to breast cancer diagnosis and the HRs of overall mortality (p < 0.001) [21]. Indeed, they found that mortality was almost 60% higher in women with PABC diagnosed at 12 months after delivery (HR = 1.59, 95% CI 1.30–1.82) but not significantly different at 70 months after delivery (HR = 1.14, 95% CI 0.99–1.25) compared with nulliparous women. Based on these data, several authors have suggested including patients diagnosed up to 5–10 years postpartum in the definition of PABC to capture the increased risk [19,21,25]. This suggestion is supported by data on postpartum-PABC patients who have worse survival rates and who are more than twice as likely to develop metastases than women with true PABC, and nearly three times as likely to develop metastases and die from breast cancer than nulliparous women [17,19,26,27]. This increased risk could be explained by the involution of the mammary gland corresponding to a period of lymphatic growth and remodeling with subsequent induction of immunosuppression and lymph angiogenesis in the tumor microenvironment [19,25].

In our CALG database, more than a quarter (27.1%) of the entire PABC population had TNBC. This finding is similar to the rate reported in the literature with a prevalence of TN-PABC ranging from 17% to 48% [[28], [29], [30], [31], [32], [33]]. This proportion is also similar to that observed in non-pregnant young women [34,35]. The TNBC subtype has the worst prognosis in terms of survival [36]. Women with TNBC are more likely to experience relapses and have higher death rates compared with women with other breast cancer subtypes (33.9% vs 20.4% and 42.2% vs 28%, respectively) [33]. Indeed, the clinical characteristics of TN tumors include a larger tumor size, a higher fraction of high-grade tumors, and an increased risk of distant recurrence [[37], [38], [39]]. Initially, the two groups of patients of our population with TNBC differed significantly before PMS analysis – patients in the PABC group were younger, with larger tumors, and more lymph node involvement – in accordance with previous studies on PABC and which explains the poor global prognosis in this setting [17,[40], [41], [42], [43]]. After using PSM analysis to reduce biases and restore comparable groups for tumor size and node involvement, we found that pregnancy per se was not a factor of poor prognosis. Boudy et al. also used PSM to compare the prognosis of the entire PABC CALG population and confirmed the absence of difference in survival between women with and without PABC [44].

In the last decade, the treatment of TNBC has significantly evolved both in terms of neoadjuvant treatment and metastatic disease management [45]. Although cytotoxic chemotherapy remains the mainstay of therapy, the thorough review of biomarkers in TNBC has opened up the possibility of more targeted and effective treatments such as the incorporation of checkpoint inhibitors and poly (ADP-ribose) polymerase (PARP) inhibitors [45]. In our study, the treatment of patients with TN-PABC was similar to that of TN-non-PABC patients except for dose-dense chemotherapy which is not recommended in pregnant women.

Additional issues are the modalities of TN-PABC patient treatments. More than two-thirds of TN-PABC patients received neoadjuvant chemotherapy and 25.4% adjuvant chemotherapy. Previous studies have shown that chemotherapy is not advisable during first trimester as it may induce miscarriage and teratogenic effects [40,46], whereas chemotherapeutic agents can be safely administered from the beginning of the second trimester to the end of pregnancy without risk of fetal malformation [17]. The administration of molecules usually used for breast cancer during pregnancy (taxanes and anthracyclines) has been explored in several studies and no evidence of fetal toxicity has been shown [[47], [48], [49], [50]]. However, chemotherapy during pregnancy has been associated with an increased risk of intra-uterine growth restriction and preterm labor [51]. In fact, in a multicentre cohort study of 447 patients with breast cancer during pregnancy recruited between April 2003 and December 2011, Loibl et al. reported a premature delivery rate of 50% [26]. Nonetheless, no significant difference in the frequency of premature delivery was found between patients given (45.9%) or not given (54.1%) chemotherapy during pregnancy. This rate is consistent with the one reported by de Haan et al. in a multicentre cohort study of 1170 patients diagnosed with primary invasive cancer during pregnancy between January 1, 1996 and November 1, 2016 [52]. In their study, 462 out of 1170 patients (39%) had breast cancer; 54% of them were given chemotherapy during pregnancy. Obstetrics outcomes was known for 428 out of 462 patients (92.6%); preterm birth occurred in 184 out of 428 patients (43%) [52]. In our study, 41 out of 59 TN-PABC patients (69.5%) had chemotherapy during pregnancy. Only seven out of 48 patients (14.6%) gave birth prematurely; all of them had chemotherapy during pregnancy. This discrepancy can be explained by the fact that, in our study, the inclusion period is more recent than in the other two studies. Indeed, the reassuring data on antenatal chemotherapy published a few years ago [49,50], has led to the tendency to continue chemotherapy for longer during pregnancy and a decrease in the frequency of iatrogenic premature delivery for the benefit of the child. Although the rate of preterm birth in our study is much lower than those reported in the literature, it remains higher than the prematurity rate in France estimated at 7.5% in 2016 [53]. The remaining risk results in both maternal and fetal leukopenia, which explains why chemotherapy is not administered after 35 WG so as to avoid infectious complications after delivery. International guidelines recommend that women with TN-PABC undergo the same surgical management as women with TN-non-PABC, as reflected by the similar surgical management for both groups in our study [54]. Total or partial mastectomy is feasible at any time during pregnancy [41,54]. In our study, a trend for a higher rate of axillary lymphadenectomy (67.8%, p = 0.06) was noted in the TN-PABC group probably linked to a higher prevalence of nodal involvement. The feasibility of a sentinel lymph node procedure using technetium-99 has been validated in pregnancy, with a calculated radiation dose to the fetus below the threshold dose of teratogenic effects [[55], [56], [57]]. Thus, specific guidelines for PABC are required including the use of a sentinel lymph node technique in first intention if indicated [41,54].

Some limits of the present study deserve to be underlined. First, although based on a prospective database, the retrospective nature of the study cannot rule out all biases. Patients with HER2 score 1+ and with a possible positive FISH test were included, as well as patients with low hormone receptor (HR) expression (1%–9%). Second, the low sample size, linked to the low incidence of the disease, could be a source of misinterpretation. Third, although we used a PMS analysis, there is a risk of bias linked to the epidemiological characteristics of women with TN-PABC. The median age at diagnosis in our population of women with TN-PABC was 33.8 years (IQR 30.0–37.0) which was significantly lower than that of patients with TN-non-PABC (35.7 years; IQR 32.0–39.0). Previous studies have underlined that breast cancer diagnosed at a young age is correlated with lower survival rates and higher recurrence rates when compared with older patients [34,58,59]. Indeed, Liedtke et al. found a significant correlation between age at diagnosis and overall survival, disease-free survival and distant disease-free survival in five age cohorts (≤30, 31–40, 41–50, 51–60 and >60 years) including 1732 patients with primary TNBC [58]. In their study, the unfavorable effect of young age at diagnosis on disease-free survival was independent of tumor diameter, tumor grade and nodal status. They observed the largest absolute difference between patients younger than 40 and patients aged 41 or older. Although there is a significant difference in age in our study after PSM (p = 0.03), the median age at diagnosis was in both groups less than 40 years and only two years of difference was noted, which does not seem to modify the prognostic factor.

Fourth, it is important to note that the vast majority of patients with TN-PABC diagnosed during the first trimester of pregnancy did not continue their pregnancy. Our conclusions are thus restricted to patients managed during the second and third trimesters of pregnancy. Although data in the literature are reassuring on the safety of chemotherapy during pregnancy and that all the newborns were in good health, the lack of data on their follow-up and, in particular, on the screening of potential complications is a major limitation. Fifth, data from the TN-PABC group were pooled from different hospitals belonging to the CALG network, whereas all the TN-non-BAPC patients came from Tenon Hospital only. This could explain the longer follow up of patients in the TN-non-PABC group (four times longer than for the TN-PABC group). This difference should make us remain cautious about the interpretation of the results. Many studies have shown an association between pathological complete response (pCR) and good prognosis in TNBC [[60], [61], [62]]. The analysis of the pCR would have been a way to overcome the significant difference in follow-up between the two groups. However, as the majority of patients in the TN-PABC group are from different hospitals belonging to the CALG network, we only had pCR for 15 out of 59 patients, which made us unable to analyze this data. This underlines the need for practitioners to consult the CALG network so that both mother and obstetrical outcomes can be captured and monitored.

In conclusion, although the TN subtype is associated with a poor prognosis in pregnant patients due to the advanced stage at diagnosis and high lymph node involvement, our PSM case-control study shows that pregnancy per se does not worsen the prognosis in terms of RFS and OS in patients with TN-PABC.

## Funding

This study did not received any grant.

## Declaration of competing interest

The authors have no conflict of interest to declare.
